# General Practice and the Community: Research on health service, quality improvements and training. Selected abstracts from the EGPRN Meeting in Vigo, Spain, 17–20 October 2019: All abstracts of the conference can be found at the EGPRN website https://www.egprn.org/page/conference-abstracts

**DOI:** 10.1080/13814788.2020.1719994

**Published:** 2020-03-04

## Introduction to the theme of the conference

In the EGPRN’s research agenda, the community orientation domain was defined and promoted.

‘Community’ includes health care professionals and patients who, using their individual, family and social capacities, become involved in community processes to promote positive changes in their health. In strengthening the community, non-health sectors also play an important role. Professionals move from a predominant or exclusive role in decision-making, to a greater role in facilitating and collaborating in decision-making, empowering the participation of people in communities.

Our shared objective for this conference was to stimulate reflection on what we do and what we want to achieve in terms of community participation.

On behalf of the Organising Committee

Dr. Ana Clavería

