# Supplementary material for: General Practice and the Community: Research on health service, quality improvements and training. Selected abstracts from the EGPRN Meeting in Vigo, Spain, 17–20 October 2019: All abstracts of the conference can be found at the EGPRN website https://www.egprn.org/page/conference-abstracts
Source: Eur J Gen Pract. 2020 Mar 4;26(1):42–50. doi: 10.1080/13814788.2020.1719994 (PMC7067172; doi:10.1080/13814788.2020.1719994)
Supplement: Related texts

# Abstract: Community participation in primary healthcare

**Published:** 2020-03-04

**Authors:** Anne MacFarlane

**Affiliations:** Graduate Entry Medical School (GEMS), University of Limerick, Limerick, Ireland

## Abstract

Community participation in primary healthcare is enshrined in international policies since the 1970s and has been re-emphasised since then, most recently in the 2018 WHO Astana Declaration (https://www.who.int/primary-health/conference-phc/declaration). The concept comes from a social justice perspective. It emphasises that the participation of communities who experience poverty and social exclusion is essential to the development of primary health care services shaping these services and making them relevant to those with the greatest need. This is important if we are to address the well-documented Inverse Care Law.

There is, however, a translational gap between policy and practice. The stability of policies for community participation in primary healthcare is patchy. The implementation of policies into conventional ways of working is patchy. Where implementation has occurred, the coverage of community participation initiatives can be patchy – not all community members are involved. The literature shows a pattern of exclusion whereby so-called ‘hard to reach’ groups are not adequately involved in primary healthcare decision-making. This is the case for refugees and migrants who arrive to settle and integrate into host countries in Europe. The recent WHO Strategy and Action Plan for Refugee and Migrant Health (2016; http://www.euro.who.int/en/health-topics/health-determinants/migration-and-health/publications/2016/strategy-and-action-plan-for-refugee-and-migrant-health-in-the-who-european-region) is a call for action to disrupt this pattern of exclusion and improve the health of refugees and migrants.

Drawing on the rich tradition of participatory health research is a valuable way forward because it provides important concepts, tools and techniques for research that is more inclusive and primary care practice. This presentation will describe innovative examples of success in family practice settings from around Europe. These have brought together refugees and migrants with primary care stakeholders and enabled them to work together to introduce and sustain changes in clinical practice. This evidence can be used to guide and strengthen community participation in primary healthcare, for all.

# Abstract: Looking for value-based community health

**Published:** 2020-03-04

**Authors:** Jordi Varela

**Affiliations:** a Epidemiology and Statistics, CESAM, Paris, France;; b Hospital Management, ESADE, Barcelona, Spain

## Abstract

What are we talking about when we talk about value?

In 2006, Michael Porter and Elizabeth O. Teisberg published; *Redefining Health Care, Creating Value-Based Competition on Results*, Harvard Business School Press. Affirming that payers and providers, including doctors and nurses, are very concerned in demonstrating that they work a lot, and very little, or nothing, in assessing what their work contributes to the health of people and communities.

Michael Porter is famous in the business world for his work on competitiveness based on the value of products and services. He has introduced this concept in the provision of health services, all summarised in a phrase: Health systems should seek to obtain the maximum possible value for the health of people for every dollar they spend. However, to define the value in healthcare, the patient must be introduced into the equation, so, in Porterian terms, the value is the perception that people have about clinical effectiveness and the costs of therapeutic processes.

Clinical effectiveness is measurable from epidemiology (to be readmitted to a hospital fewer times or living longer); value, on the other hand, is reflected by people’s experience. We need to ask questions such as do patients with advanced diseases want to live longer, or they want to enjoy the highest quality of life possible. Depending on the response, we can develop different delivery models.

What is value-based healthcare?

According to NEJM Catalyst, Value-based healthcare is a healthcare delivery model in which providers, including hospitals and physicians, are paid based on patient health outcomes (https://catalyst.nejm.org/doi/full/10.1056/CAT.17.0558). Under value-based care agreements, providers are rewarded for helping patients improve their health, reduce the effects and incidence of chronic disease, and live healthier lives in an evidence-based way.

How to achieve a Value-Based Healthcare Model

The following six drivers are the key to make a primary health care system a value-based healthcare model:

1. Prioritising patient-centred care.

2. From clinical pathways to care delivery value chains.

3. Promoting the right care and reducing medical overuse.

4. Turning a fragmented model into another integrated model.

5. Creating the enabling environment for healthcare transformation.

6. Fostering community health.

How to develop a Value-Based Community Health

Michael Marmot states that if the determinants of health are mostly social, solutions must also be social, so to improve the quality of community life, political systems require economic, housing, education, security and infrastructure programs (Am J Public Health. 2014;104:S517–S519). Nevertheless, the healthcare system must know how to adjust resources according to the social circumstances of each community and to understand how to provide a health-oriented vision of all the social programs. On the other hand, community health is an intervention model that aims to improve the health of a defined community that should operate from primary care services to adjust their actions to the social reality of each territory.

# Abstract: ‘It’s a double-edged sword:’ Translation and cultural adaptation of a prostate cancer screening decision aid: Qualitative study

**Published:** 2020-03-04

**Authors:** Sofia Baptista, Bruno Heleno, Marta Pinto, Bruna Guimarães, Diogo China, João Pedro Ramos, Andreia Teixeira, Kathryn Taylor, Carlos Martins

**Affiliations:** a Faculdade de Medicina, Universidade do Porto, Porto, Portugal

## Abstract

**Background:** Screening for prostate cancer remains controversial, implying a trade-off between benefits and harms, and a shared decision-making process has been advocated. Decision aids are evidence-based tools that improve decision quality. For limited-resource countries, translating and making cultural adaptations to high-quality decision aids is a reasonable alternative to developing new ones.

**Research question:** We aimed to translate and culturally adapt an English language patient decision aid addressing prostate cancer screening, so that Portuguese men can use it.

**Methods:** We followed the European Centre for Disease Prevention and Control’s (ECDC) five-step, stakeholder-based approach to adapting health communication materials: (1) selection of materials and process coordinators; (2) early review; (3) translation and back translation; (4) comprehension testing with cognitive semi-structured interviews; (5) proofreading. Cognitive interviews were conducted with 15 men, ages 55–69, from the Oporto district local community to refine the decision aid after its translation. Content analysis was performed using Ligre™ software.

**Results:** Five main themes are presented: informational content, information comprehension, socio-cultural appropriateness, feelings and primary message, and personal perspective concerning prostate cancer screening. For each theme, illustrative quotes extracted from men’s interviews are presented. Most men found the translated version of the decision aid to be clear, comprehensive and appropriate for its target population, albeit some suggested that medical terms could be a barrier. The data collected from men’s interviews allowed the researchers to clarify concepts and expand existing content.

**Conclusion:** The final version of the decision aid can be used in the real world clinical setting and our ECDC based approach can be replicated by other workgroups to translate and culturally adapt decision aids.

# Abstract: Geriatric community care for the future – The Borgholm model

**Published:** 2020-03-04

**Authors:** Hans Thulesius, Åke Åkesson

**Affiliations:** a R&D Kronoberg, Clinical Sciences, Linnaeus University, Växjö, Sweden

## Abstract

**Background:** Geriatric care needs to be increased with growing elderly populations. The Borgholm jurisdiction in the Baltic Island of Öland (Kalmar region) has an older than average senior population and had difficulties recruiting primary care physicians (PCPs) resulting in high elderly hospital care consumption.

**Research question:** Could a new model of geriatric care be able to decrease the hospital care needs of Borgholm as compared to the rest of Kalmar region?

**Methods:** A new model of care was developed in Borgholm 2016–2017 where the PCP list was limited to 1000 patients, daily slots for PCP home care visits could be booked by community nurses or ambulance nurses and PCPs had daily anticipatory care planning contacts with Kalmar hospital staff.

**Results:** Between 2014 and 2018, Borgholm home care patients >75 years old increased by 70% vs. a 2% decrease for the rest of Kalmar region. Similarly, Kalmar emergency department visits decreased by 19% in Borgholm vs. 9% increase for the rest of Kalmar. Also, Kalmar hospital care episodes decreased 7% in Borgholm vs. 13% increase for the rest of Kalmar; Kalmar hospital outpatient visits decreased 8% in Borgholm vs. 21% increase for the rest of Kalmar; total care consumption for >75 years old decreased 4% in Borgholm vs. a 10% increase for the rest of Kalmar region.

**Conclusion:** A new geriatric care model consisting of a comprehensive collaboration between strengthened primary care and community care, hospital care and ambulance care was associated with a reduction in total care consumption for senior citizens in a rural Swedish jurisdiction.

# Abstract: Changes in the social support, emotional state and quality of life, after a program of physical activity in the elderly people: Clinical trial multi-centre randomized

**Published:** 2020-03-04

**Authors:** Anna Ruiz, Glòria Sauch, Jacobo Mendioroz, Pere Roura, Anna Sabata, Irene Cornet, Isabel Gómez, Àngels Casaldàliga, Carme Saldaña, Josep Vidal, Montserrat Fusté, Carme Boix, Berta Rodoreda, Anna Ramirez

**Affiliations:** a Catalan Institute of Health, Sant Joan de Vilatorrada, Spain

## Abstract

**Background:** Social isolation, loneliness and anxiety-depressive states are emerging health conditions in the elderly.

**Research question:** To assess whether a 4-month programme of physical activity in a group improves the emotional, social and quality of life situation in a sample of subjects over 64 years old people.

**Methods:** Multi-centre randomized clinical trial of two groups. *Study population*: Patients older than 64 years assigned to three primary care teams from different locations. *Inclusion criteria*: Submit a score <32 on the DUKE-UNC-11 social support scale, or >12 on the Beck Depression Scale, or >10 on the Generalized Anxiety Scale (GAD-7), at the start of the study. The intervention group participated in a group physical activity program for 4-months that consisted of progressively walking sessions two days a week, 60–150 minutes long depending on the physical condition of each participant.

**Results:** Enrolled were 94 patients who met the inclusion criteria. Mean age was 74 years (SD 5.18) and 76.6% were women. No significant differences were found at the beginning of the study between the two groups in relation to the outcome of the scales evaluated. Once the intervention was completed, improvement in the quality of life and social support was detected in the intervention group (*p*<.05). Both groups improved the depression and anxiety clinic but the improvement in the participants of the intervention group was higher. Those with initial depression improved 8.6 points on the scale, compared to the control that improved 3.3 points, with the final average of 17.4. Those who presented initial anxiety improved 8 points (final average: 7.5 points, cut-off point for the diagnosis of anxiety 10), compared to the control that improved 5.1 points.

**Conclusion:** The results of this study indicate that the program developed has positive effects on improving the quality of life, social support and depression and anxiety clinic.

# Abstract: Prevalence and care for patients with dementia in primary care

**Published:** 2020-03-04

**Authors:** Katarina Stavrikj, Marta Tundzeva, Katerina Kovachevikj, Ljubin Sukriev

**Affiliations:** a Medical Faculty, Center for Family Medicine, Skopje, Macedonia

## Abstract

**Background:** Primary health care, the general practitioner, plays a critical role for early identification and care of patients with dementia. Early diagnosis of dementia allows starting therapy and improving the quality of life of the patients.

**Research question:** To estimate the prevalence and care of patients with dementia in North Macedonia.

**Methods:** Forty-six general practitioners (GPs) surgeries from 20 cities in Macedonia took part in the project. All individuals age over 65 years with a diagnosis of dementia were identified from GP electronic disease registers.

**Results:** Based on the diagnosis, 450 (3.5%) patients were identified from a total population of 12,926 over 65s. The most common dementia was Alzheimer’s dementia 294 (65.3%) followed by vascular dementia 27.11%. The average age of respondents in the study was 77.5 ± 8.2 years, with 50% patients under the age of 79 years, 65.6% were female and 68.4% were with elementary school. In the entire sample, most of the patients diagnosed with dementia 195 (43.3%) said they lived with another family member. The most common risk factor was hypertension (85.1%), followed by stroke/ transitory ischemic attacks (29.3%) and equal percentage, i.e. 26.4% of patients had high levels of cholesterol and diabetes. To 242 (53.8%) acetylcholinesterase inhibitors were prescribed (donepezil, rivastigmine, galantamine), 77 (17.1%) memantine, while 247 (54.9%) another OTC therapy. 227 (50.4%) reported that they did not receive treatment. An additional analysis of the reasons for not receiving treatment was made on this sample of patients who did not receive treatment. It was found that in the majority of these patients (more than 50%) the reason for not receiving therapy was that it was not prescribed, in 142 (62.6%).

**Conclusion:** This is the first national representative study of dementia prevalence in North Macedonia. Those data can provide information for healthcare needs people with dementia.

# Abstract: Hotel housekeepers: Working conditions and health – A mixed-methods study

**Published:** 2020-03-04

**Authors:** Joan Llobera, Xenia Chela, Maria Clara Vidal, Oana Bulilete

**Affiliations:** a Primary Care Mallorca, Ibsalut, Research Unit PHC, IdISBA, Palma de Mallorca, Spain

## Abstract

**Background:** Tourism represents 45% gross domestic product in Balearic Islands. Working as a hotel housekeeper (HH) has been associated with important morbidity, especially musculoskeletal, chronic pain, a significant number of sick leaves, a high consumption of medication, poor psychological well-being and worse quality of life.

**Research question:** Explore perceptions and opinions regarding the HH’s work and health problems. Estimate and evaluate HH’s health determinants, the exposition to several occupational risk factors, their lifestyles and health problems and their quality of life.

**Methods:** Design: mixed methods: (1) exploratory qualitative study (QS) including 10 semi-structured interviews and six focus groups; (2). descriptive study (DS): individual interviews and clinical medical records. *Inclusion criteria*: older than 18 years, had worked during the last summer season in the Balearic Islands. *Analysis*: QS: transcription and content analysis; DS: descriptive statistical analysis.

**Results:** QS: Identified positive aspects of their work: timetables, relationship with co-workers, attending clients. Highlighted negative aspects: working conditions, hard physical workload, stressful duties and insufficiently rewarded. HH associated their health problems with their work; coping strategies: self-medication or visiting their general practitioner. DS: 1.043 HH included. Mean age 43.3 years, mean working years as HH 10.7 years. Mean rooms/day: 18.1 (±6.5); mean beds/day: 44.6 (±20.7). HH reported often pain during the last summer season: 68.2% (IC 95% 65.3–71.0) low back pain; 60.9% (IC 95% 57.8–63.8) wrist and hands; 55.3% (IC 95% 52.2–58.3) cervical. 41.6% and 35.1% self-reported regular and poor health status, respectively.

**Conclusion:** HH perceived hard and stressful working conditions, partly justified by the number of rooms and beds made per day. They also perceived health problems related to their work. HH frequently reported pain during the last summer season. Moreover, they perceive regular or poor health status, weaker than women from the same social class do.

# Abstract: Effectiveness of a gender-based violence intervention in primary health centres in Vigo Area, Spain

**Published:** 2020-03-04

**Authors:** Aránzazu Dorrego, Laura Otero Gómez, Pedro Jose Otero Rivas, Beatriz Pérez Sánchez

**Affiliations:** a Vigo Family and Community Medicine Teaching Unit, EOXI Vigo, Vigo, Spain

## Abstract

**Background:** Gender-based violence (GBV) is a public health and human rights issue, being highly prevalent (12–51%), repetitive and having a severe impact on women’s health, with a high sanitary and social cost. Primary care has a key role in detection and management. There is low detection and delay in diagnosis. There is a lack of preparation to recognize abuse, especially in the approach and action after detection. Greater awareness and sensitization is required.

**Research question:** Can a brief specific training intervention in GBV imparted to primary health care professionals in their primary health centre increase knowledge, improve attitudes and skills?

**Methods:** A cluster-randomized clinical trial was carried out in Vigo area primary health centres with at least 20 health care professionals. A basal evaluation was made through a validated inquiry (PREMIS), which they had to retake after three months. In the intervention centres, a clinical session was imparted. *p*<.05.

**Results:** Out of 264 primary health care professionals, 145 participated. There was a 63.5% loss out of 145 professionals. A statistically significant difference was detected in the field of knowledge, increasing an average of two points on a scale from 0 to 5 in these aspects: how to make appropriate questions; connections between GBV and pregnancy; why do not they leave their partners; risk determination and phases of GBV. There was also a decrease in the idea that if the patient does not recognize gender violence, there is very little that can be done. No significant differences were detected in the detection and follow-up.

**Conclusion:** Significant differences were found in the knowledge and attitude sections after performing the intervention to the professionals. The results support the implementation of continuous brief training on GBV in primary care.

# Abstract: Engaging stakeholders in community-oriented research: Experiences from European research programmes

**Published:** 2020-03-04

**Authors:** Marilena Anastasaki, Christos Lionis

**Affiliations:** a Clinic of Social and Family Medicine (CSFM), University of Crete, Heraklion, Greece

## Abstract

**Background:** Community participation is essential for effective implementation of research programmes in primary healthcare (PHC) but also appropriate interpretation of results and optimal delivery of subsequent care. Stakeholder engagement undertaken under defined and evaluated frameworks may be key for the establishment of concrete collaboration and communication between communities and other parties involved in research. This abstract aims to report on community and stakeholder engagement methodologies, plans and activities of European research projects conducted in Crete, Greece.

**Research question:** Could a consensus be reached regarding the methods and tools for enhancing stakeholder engagement in community-oriented PHC research?

**Methods:** Examined programmes included RESTORE (FP7), FRESH AIR (Horizon2020) and VIGOUR (Health Programme). Identified methodologies included Normalisation Process Theory, Participatory Learning and Action, Five Steps of Stakeholders’ Engagement, establishment of Stakeholder Engagement Groups under the 9 C’s model (commissioners, customers, collaborators, contributors, channels, commentators, consumers, champions, competitors) and Structured Democratic Dialogue. These were implemented to a range of stakeholders, including community members, patients, migrants, Roma populations, healthcare professionals and policy-makers. Qualitative research (focus groups, individual interviews) and Thematic Content Analysis were used for design and analysis of engagement activities.

**Results:** In RESTORE, migrants and other stakeholders selected guidelines and training supporting cross-cultural communication in PHC consultations, based on their own needs and expectations. Community members, healthcare professionals and healthcare authorities were actively involved in FRESH AIR by identifying local priorities and contextual factors for designing project interventions, providing access to communities and supporting dissemination of project achievements. In VIGOUR, multidisciplinary stakeholders were brought together and formulated a joint ambition statement for the future of integrated care in Crete.

**Conclusion:** Various stakeholder engagement methods with documented effects are currently available. Their systematic identification, appraisal, synthesis and consolidation may serve with enhancing community participation in PHC, sustaining research results and translating findings into appropriate actions.

# Abstract: iCAREdata: A scientific research database on out-of-hours primary care

**Published:** 2020-03-04

**Authors:** Hilde Philips, Stephaan Bartholomeeusen, Paul Van Royen, Roy Remmen, Samuel Coenen, Annelies Colliers, Stefan Morreel, Veronique Verhoeven

**Affiliations:** a Center for General Practice Atwerpen, University of Antwerp, Antwerp, Belgium

## Abstract

**Background:** Out-of-hours (OOH) primary care is a topic of great interest in European countries. Reasons for this are similar across borders: to guarantee continuity of care with decreasing numbers of health care workers and to guard equity in OOHcare for all patients. In OOHcare research, valid and accessible research data are needed to fill the knowledge gap. iCAREdata aims to offer valid and immediately available information from OOHcare.

**Research question:** How feasible is it to collect, store and link data of different OOH services in Belgium and to improve data quality registration?

How useful are aggregated data to inform stakeholders, to evaluate (the quality of) services in OOH care and the effects of interventions?

**Methods:** As a first achievement, data flows, encryption and encoding were carefully designed and implemented. Solid cooperation with the federal eHealth web services as a trusted third party was crucial. Ethical approval and approval by the data protection authority was obtained. Clear agreements were established concerning access control. A strict code of conduct was agreed upon. A steering committee was established to guard the procedures.

**Results:** First data were collected in 2015. iCAREdata now receives ±3000 unique patient contacts per weekend, spread over 14 general practice cooperatives, and covering about a quarter of the Flemish population. Aggregated data, directly processed, are provided weekly on https://icare.uantwerpen.be. This portal site offers an overview of, among others, the latest diagnostics, drug prescriptions and workload. iCAREdata project also collects data from emergency departments in hospitals and community pharmacists and link them to evaluate further OOH primary care.

**Conclusion:** Developing a research database on OOHcare is feasible. The iCAREdata project succeeds in an automated output every week, offering insights on the evolution of morbidity, services and effects of interventions. Careful validation and interpretation of the data is a crucial ongoing challenge.

# Abstract: Triaging and Referring In Adjacent General and Emergency departments (the TRIAGE-trial): Preliminary results of a cluster randomised controlled trial

**Published:** 2020-03-04

**Authors:** Stefan Morreel, Veronique Verhoeven, Hilde Philips

**Affiliations:** a Department of General Practice, University of Antwerp, Antwerpen, Belgium

## Abstract

**Background:** Patients who might also go to the general practitioner (GP) frequently consult emergency departments (ED). This leads to decreased efficiency, high workload at the ED and additional costs for both government and patient.

**Research question:** The primary outcome is the proportion of patients who enter the ED and are handled by the GP after triage. *Secondary outcomes*: Referral rate to the ED by the GP, proportion of patients not following the triage advice, compliance of the nurse to the triage-instructions and health insurance expenditures. Furthermore, facilitators and barriers will be studied and an incident analysis will be performed.

**Methods:** This is a randomised controlled trial with weekends serving as clusters. Patients presenting at the ED during OOH are triaged and allocated to either ED or GP by a trained nurse using an extension to the Manchester Triage System (MTS). During control clusters, all patients remain at the ED. Data are collected using a database for OOH care (iCAREdata).

**Results:** So far, 296 out of 2733 (11%) patients were allocated to the GP. Two-thirds (194) of these patients did go to the GP leading to a primary outcome of 7% for 14 intervention weekends. Only eight patients were referred back to the ED. Compliance of the nurse to the extended MTS was 93%, in 6% of the cases the nurse chose ED instead of GPC and in less than one percent GPC instead of ED. The nurses chose higher urgency categories and more discriminators, leading to the GP during intervention clusters. Using an automated system, these results are updated weekly, on our poster, we will show more results that are complete.

**Conclusion:** These first results reveal a low efficiency but a high safety of the intervention. More prolonged data collection combined with a process analysis and cost efficiency study is necessary before definitive conclusions can be drawn.

# Abstract: Adherence to oral anticoagulant medications, are all medications equal?

**Published:** 2020-03-04

**Authors:** Michal Shani, Doron Komaneshter, Alex Lustman

**Affiliations:** a Clalit Health Service, Family Medicine, Mazkekret Batya, Israel

## Abstract

**Background:** Oral anticoagulants (OAC) reduce the risk for stroke and death from all causes in patients with non-valvular atrial fibrillation (NVAF).

**Research question:** To explore adherence rates to OAC among patients with NVAF and to compare head-to-head adherence rate of different medications in long-term chronic use.

**Methods:** We conducted a population-based cohort study Clalit Health Services, Israel. All patients, 30 years and over, with a diagnosis of NVAF before 2016 and were treated with OAC were included. We included patients that filled at least one prescription per year in the three consecutive years 2016–2018. We analysed all prescriptions that were filled for the medications from 1 January 2017 to 31 December 2017. We considered purchasing of at least nine monthly prescriptions during 2017 as ‘good medication adherence.’

**Results:** Twenty-six thousand and twenty-nine patients with NVAF who were treated with OAC were identified. Ten thousand and two hundred and eighty-four (39.5%) were treated with apixaban, 6321 (24.3%) were treated with warfarin, 6290 (24.1%) were treated with rivaroxaban 3134 (12.0%) were treated with dabigatran. Rates of good medication adherence were 88.9% for rivaroxaban, 84.9% for apixaban, 83.6% for dabigatran and 55.8% for warfarin (*p*<.0001). Good adherence with OAC was associated with lower LDL cholesterol and glucose levels. Advanced age was associated with higher adherence rates (*p*<.001). SES was not associated with medication adherence.

**Conclusion:** Adherence rates to DOAC among patients with NVAF are high and are higher than the adherence rate to warfarin. It should be taken into consideration when choosing OAC treatment for NVAF.

# Abstract: Hefestos score: Short-term prognosis stratification of heart failure decompensations in primary care

**Published:** 2020-03-04

**Authors:** Miguel Angel Muñoz, Jose-Maria Verdú-Rotellar, Hélène Vaillant-Roussel, Hans Thulesius, Radost Assenova, Peter Torzsa, Durdica Lazic, Liam Glynn, Josef Woebkenberg, Jacopo Demurtas, Rosa Abellana

**Affiliations:** a Departament de Salut | Generalitat de Catalunya, Idiap Jordi Gol & semFYC, Barcelona, Spain

## Abstract

**Background:** More than half of decompensations of heart failure are attended in primary care setting. No score that helps to ascertain the short-term prognosis in these patients.

**Research question:** To develop and validate a short-term score (30 days) to predict hospitalizations or death in patients attended in primary care as a consequence of decompensation of heart failure, based on variables easily measurable in primary care setting

**Methods:** Prospective multinational cohort study including patients treated because of a heart failure decompensation in primary care setting. There were a derivation (Spain) and a validation cohort (nine European countries).

**Results:** The derivation cohort included 561 patients, women were 56%, mean age was 82.2 (SD 8.03) years and 31.5% of patients were hospitalized or died in the first month. In the validation cohort, 238 patients were included, women were 54%, mean age was 79.0 (10.4) years and 26.9% of patients were hospitalized or died in the first month. According to the multivariate models, sex, age, hospital admission due to heart failure the previous year, and a heart rate greater than 100 beats/minute, orthopnoea, paroxysmal nocturnal dyspnoea, NYHA functional stage III or IV, saturation of oxygen lower than 90% or an increase in the dyspnoea at the consultation with the General practitioner were included in the HEFESTOS-SCORE. The multivariate model including these variables showed a good calibration (Hosmer–Lemeshow *p*=.35) and discrimination (AUC 0.81, 95% CI 0.77–0.85). In the validation cohort, the model presented an adequate external validation with good calibration (Hosmer–Lemeshow *p*=.35) and discrimination (AUC 0.74, 95% CI 0.67–0.82).

**Conclusion:** The HEFESTOS-SCORE, based on clinical and demographical variables easily measurable in primary care is a useful tool to stratify the short-term hospitalization and mortality in patients attended because of a heart failure decompensation.

# Abstract: Barriers and facilitators for cardiovascular primary prevention in a French rural deprived area: Exploration of caregivers’ and patients’ experiences by using qualitative interviews

**Published:** 2020-03-04

**Authors:** Delphine Le Goff, Michele Odorico, Sophie Lalande, Jérémy Derriennic, Jean Yves Le Reste

**Affiliations:** a CCU Département de Médecine Générale, Faculté de Médecine, Université de Bretagne Occidentale, Brest, France

## Abstract

**Background:** Cardiovascular diseases (CVDs) are the first mortality cause worldwide with 17.5 million death in 2012. Spices (Scaling-up Packages of Interventions for CVD prevention in selected sites in Europe and Sub-Saharan Africa) gathered five countries around CVD primary prevention interventions, especially for populations with low access to prevention and health care system. In France, a rural area where people were more deprived and with a low settlement of general practitioners (GPs) fitted with the project.

**Research question:** What are the barriers and the facilitators for cardiovascular primary prevention implementation from caregivers and patients’ point of view of a deprived rural area?

**Methods:** Semi-structured interviews were conducted until theoretical saturation of data. Purposive samplings of GPs, patients, patients’ families, nurses and pharmacists were designed. Five interview guides explored cardiovascular prevention, cardiovascular health promotion in the setting, actors of CVD prevention, capacities for CVD prevention, patients’ and healthcare professionals’ representations, barriers and facilitators in implementing CVD prevention, possible solutions. Guides were adapted concurrently to the analysis. A blinded thematic analysis and a mind-mapping were achieved for each group.

**Results:** Thirteen GPS, 11 pharmacists’, 14 nurses, 12 patients’ and 12 patients’ family members’ interviews were achieved. Professionals highlighted a disconnection between them and national prevention programs, lack of time, payment and training for CVD prevention. Countryside was either protective or aggressive regarding CVD risk balancing gardening and space against isolation and lack of structures. GPs had poor connections with the community. Patients described their recklessness and feeling of invulnerability until their CVD appeared. Families could be a barrier to CVD prevention and lifestyle change. Risky behaviours were handed down from one generation to another.

**Conclusion:** Innovative interventions for Spices should focus on these community specificities and individual behavioural strategies in contrast with the six national plans addressing CVD in France. These plans solely concentrate on dissemination of prevention messages and knowledge, which is of little use according to this survey.

# Abstract: Effectiveness of an educational and feedback intervention to reduce benzodiazepine prescriptions in primary care: A cluster randomised controlled trial: The BENZORED study

**Published:** 2020-03-04

**Authors:** Caterina Vicens Caldentey, Alfonso Leiva, Ferran Bejarano, Ermengol Sempere, Isabel Socias, Francisca Fiol Gelabert, Catalina Mateu, Santiago Alegret, Asuncion Ajenjo, Raquel Rodriguez, Silvia Folch, Marta Mengual

**Affiliations:** a Gerencia de Atención Primaria, Universitat de les Illes Balears, Calviá, Spain

## Abstract

**Background:** Despite recommendations against long-term benzodiazepine (BZD) use, they are often prescribed during months or years in primary care.

**Research question:** To evaluate the effectiveness of a primary care educational and feedback intervention targeted to general practitioners (GPs) to reduce BZDs prescriptions.

**Methods:** *Design*: A two-arm parallel cluster randomized clinical trial. *Settings*: Primary Healthcare centres from three health districts of Spain: Balearic Islands (IbSalut), Catalonia (Institut Català de la Salut; Tarragona-Reus district) and Community of Valencia (Conselleria de Salut Universal; Arnau de Vilanova llíria district). *Participants*: All GPs from the health districts included were invited to participate. Ninety percent of the GPs accepted to participate. *Intervention*: GPs received an educational two hours workshop training about the rationale for prescribing BZDs and deprescribing strategies for long-term BZD users, audit and monthly feedback about their prescription and access to a support web page with information to help them and leaflets to give to the patients. *Control group*: GPs did not receive any component of the intervention. *Outcomes*: Defined daily dose (DDD)/1000 inhabitants/year (DHD) of BZDs prescribed by GP at 12 months. Proportion of long-term BZD users (>6 months) and in patients aged 65 or more at 12 months. *Statistical analysis*: Generalized mixed linear random effect models to account for clustering at the level of healthcare centre and all analyses were based on an intention to treat principle.

**Results:** We included 749 GPs and 49 (6.5%) were lost to follow-up. Adjusted difference between groups in DHD at 12 months was −3.26 (−4.87;−1.65), *p*<.001. The differences in the proportion of long-term BZD users was −0.39 (−0.58;−0.19), *p*<.001 and in patients older than 65 was −0.87 (−1.35;−0.26), *p*=.004.

**Conclusion:** An educational and feedback intervention targeted to GPs is effective to reduce BZD prescription in primary care.

# Abstract: Implementation of a primary care educational and feedback intervention to reduce benzodiazepines prescriptions (BENZORED): A qualitative evaluation

**Published:** 2020-03-04

**Authors:** Isabel Socias, Caterina Vicens Caldentey, Ermengol Sempere, Ferran Bejarano, Haizea Pombo, Alfonso Leiva, Francisca Fiol Gelabert, Silvia Folch, Catalina Mateu, Fernando Do Pazo, Santiago Alegret

**Affiliations:** a Manacor Healthcare Centre (Ibsalut) Mallorca, Muro, Spain

## Abstract

**Background:** Despite recommendations against long-term benzodiazepine (BZD) use, they are often prescribed during months or years in primary care.

**Research question:** To determine facilitators and barriers that explain the variation in implementation of a primary care educational and feedback intervention targeted to general practitioners (GPs) to reduce BZDs prescriptions.

**Methods:** A hybrid type I clinical trial: qualitative data to evaluate the implementation outcomes.

Three health districts of Spain: Balearic Islands, Tarragona-Reus district (Catalonia) and Arnau de Vilanova llíria district (Valencia). Forty stakeholders (GPs) participated in five focus groups; they were selected based on their effectiveness of the intervention results: high (three groups) or low (two groups) and individual interviews to two GP of low efficiency. The Consolidated Framework for Implementation Research (CFIR) was used to guide collection and analysis of qualitative data. Two researchers evaluated the qualitative data of the focus groups by the Codebook and Rating Rules of CFIR, independently.

**Results:** Of the 31 CFIR constructs assessed, three constructs strongly distinguished between GPs with low versus high success of the intervention (intervention complexity, individual state of change, key stakeholders engaging), seven additional constructs weakly distinguished (adaptability, external policy and incentives, implementation climate, compatibility, relative priority, self-efficacy, formally appointed internal implementation leaders), 10 had insufficient data to assess and 11 were non-related to the success of the intervention.

**Conclusion:** We identified the constructs that explain the variation in the effectiveness of the intervention; this information is relevant to redesign successful implementation strategies focused on these constructs to implement the BENZORED intervention in health services.

# Abstract: Effectiveness of a biopsychosocial multidisciplinary intervention, by the role of fear-avoidance beliefs in non-specific sub-acute low back pain: Cluster randomized trial

**Published:** 2020-03-04

**Authors:** Romina Raczy Mas

**Affiliations:** a The University Institute for Research in Primary Health Care Jordi Gol i Gurina (IDIAPJGol), Barcelona, Spain

## Abstract

**Background:** Low back pain is a multifactorial condition with individual and societal impact. Psychosocial factors play a larger prognostic roll. Therefore, earlier multidisciplinary treatment strategy (physical, psychological and social/occupational) could be applied to search improvement in fear-avoidance beliefs with positive effect in the evolution of low back pain.

**Research question:** Evaluate the effectiveness of a biopsychosocial multidisciplinary intervention (physiotherapy, cognitive-behavioural and pharmacological therapy) through the changes in fear-avoidance beliefs (FABs), in working population with sub-acute non-specific LBP, compared to usual clinical care at 3 and 12 months.

**Methods:** A cluster randomised clinical trial, conducted in 39 Primary Health Care Centres (PHCC) in Barcelona. Participants between 18 and 65 years old (*n* = 369; control group =188, PHCC 26 and intervention group =181, PHCC 13). Control group received usual care, according to guidelines. Intervention group received usual care plus a biopsychosocial multidisciplinary intervention (sessions 10 hours/total). The main outcome was the Fear-Avoidance Beliefs questionnaire (FABQ). *Other outcomes*: Evolution to chronicity. Assessment at baseline, 3 and 12 months. Analysis was by intention to treat and analyst blinded. Multiple imputations.

**Results:** Of the 369 enrolled patients with LBP, 421 (84.0%) provided data at the three months of follow-up, and 387 (77.2%) at 12 months. Mean age of study subjects at baseline was 45.1 (SD: 10.4) years-old and 61.2% were women. At baseline, there were no differences. Both groups showed a decrease in FABQ (FAB physical and FAB-work) at three months and twelve months, with a significant difference at long-term. At FAB-physical performance, there was no significant difference over the follow-up time and at FAB-Work, a substantial difference at 12 months between groups.

**Conclusion:** A multidisciplinary biopsychosocial intervention showed a positive effect in FABs by improving fear behaviours and avoidance at work.

# Abstract: Medical student’s emotional development in early clinical experience: A model

**Published:** 2020-03-04

**Authors:** Joseph Marseille, Michel Cunin

**Affiliations:** a Faculté de médecine, General Practice, University of Lille, Lille, France

## Abstract

**Background:** Emotional experience for medical students during clinical internships is often ignored. Yet, its influence on professional skills is certain.

**Research question:** ‘What is the emotional experience of second and third-year medical students during their first clinical internship? How do they perceive the management of their experience by their supervisors?’

**Methods:** A qualitative study was conducted with 12 students in their second or third year of medical training at the University of Lille, in France, between 2016 and 2019. Interviews were carried out comprehensively for a total of 17 hours. Following a grounded theory approach, the analysis terminated when data were sufficient to offer a conclusive model.

**Results:** Emotional experience during clinical internship was rich and intense. It was most often ignored and was not taken into account in the development of professional skills. The organized management was deficient. Informal training existed: when a wilful student met a dedicated teacher. Students would have welcomed a possibility to experience intense emotions in a protective environment, and only then in an empowering environment. They expressed the same desire about early exchanges on the experiences of the internship. A modelling of the informants’ emotional experiences was realized in the form of three diagrams.

**Conclusion:** Students ask to be challenged to face patients, and then to be listened to about it. Possible interventions are trauma prevention and detection of malaise in the workplace; teaching of humanist values; providing experience and reflexivity through new pedagogical means (such as cinema, theatre, literature, writing), or relational means (such as exchange groups, companionship, solidarity commitment, immersive internships and tutoring); and training supervisors.

# Abstract: Reliability of Health Insurance claim databases to enumerate women not reached by cervical cancer screening on a 6 years follow-up in primary care

**Published:** 2020-03-04

**Authors:** Christophe Berkhout, Thibaut Raginel, Margot Badelon, Jonathan Favre, Matthieu Calafiore, Michaël Rochoy

**Affiliations:** a Department of General Medicine, Medical School, Université de Lille, Lille, France

## Abstract

**Background:** In France, cervical cancer screening by pap-smears should be conducted triennially. Screening statistics are based on the number of cytology examinations of smears reimbursed by the Health Insurance appearing in the claim databases. The percentage of screened women is lower based on these data than on declarative surveys. If surveys are overestimating the number of screened women, it is likely that claim databases underestimate it.

**Research question:** The primary objective was to determine the underestimation of screened women in claim databases. The secondary purpose was to estimate the proportion of female patients not reachable by their GP for a cervical cancer screening in an organized screening trial.

**Methods:** The population was the 6327 female patients aged 30–65 years of the 24 GP investigators of the PaCUDAHL-Gé trial. We compared the lists of their female patients that had no cytology of Pap test reimbursed during the three prior years, extracted from the Health Insurance claim databases in 2015 and 2018. We selected the patients appearing on both lists meaning they had not responded to the invitation of their GP to be screened in the trial. We searched in the GPs’ records valid reasons not to be screened (hysterectomy, history of cervical lesion, pregnancy, other conditions making screening irrelevant) or evidence of screening.

**Results:** The total number of ‘unscreened’ women in 2018 was 2731, 1737 patients appeared on both lists, 1522 could be included for analysing, 65 had been screened, 95 had hysterectomy, three had a history of cervical lesion, nine were pregnant and 10 had other conditions making screening irrelevant, 166 patients were lost to view.

**Conclusion:** Based on GPs’ records, health insurance claim databases underestimate the number of screened women by 7.6%. The percentage of patients not responding to the invitation of their GP to be screened in the PaCUDAHL-Gé trial is 24.18%.

# Abstract: Psychometric properties of the Spanish version of Hopkins Symptom Checklist-25 scale for depression detection in Primary Care

**Published:** 2020-03-04

**Authors:** Maria Rodriguez Barragan, Ana Clavería, María Isabel Fernández San Martín, Eva Peguero, Magallon Botaya, Patrice Nabbe, Jean Yves Le Reste, Miguel Angel Muñoz

**Affiliations:** a Centre d’Atenció Primària La Mina, Gerència Territorial de Barcelona, Institut Català de la Salut, Barcelona, Spain

## Abstract

**Background:** As a collaborative project of the Family Practice Depression and Multimorbidity group of European General Practice Research Network, the Hopkins Symptom Checklist-25 (HSCL-25) scale was identified as valid, reproducible, effective and easy to use. Subsequently, it has been translated and adapted to 13 languages, including Castilian. Currently, the scale is being validated in different languages.

**Research question:** What are the psychometric properties of the Spanish version of HSCL-25 (HSCL-25e) for depression detection in Primary Care?

**Methods:** HSCL-25e was administered to outpatients recruited by their physicians in six health centres involved in Spanish EIRA3 study, a trial to promote healthy behaviours in people aged from 45 to 75. Patients complimented HSCL-25 themselves. Sample size was calculated with R package (pROC). Statistical analysis: responsiveness was analysed with missing data and detecting ceiling and floor effects for the items. Principal component analysis (PCA) was done to determine the dimensions of HSCL-25e. Item-total correlation, Cronbach’s alpha (global and dimensions coefficient) and squared multiple correlation were carried out to calculate internal consistency.

**Results:** Seven hundred and sixty-nine patients out of 806 complimented HSCL-25e, 738 answered to all of the items. No patterns of missing answers were found. No ceiling effects, expected floor effect in item 18. Item 17 was the most consistent one and item 24 was the lower one. All items showed positive discrimination index for both cut-off points (1.55 and 1.75). PCA indicated two factors; 13 items corresponding to depression dimension and the other 12 items corresponding to anxiety subscale. Global Cronbach’s alpha was 0.92 (0.88 calculated for depression dimension and 0.84 for anxiety dimension).

**Conclusion:** The HSCL-25e has excellent psychometric properties when applied to Primary Care population. It has two dimensions as the original version, although the items included are not exactly the same. There are more item coincidences with the French version.

# Abstract: BIG DATA in primary care: The used algorithms have no reproducibility

**Published:** 2020-03-04

**Authors:** Jean Yves Le Reste, Eric Picel, Jeanlin Viala, Jerome Fonseca, Delphine Le Goff, Sophie Lalande, Jérémy Derriennic, Patrice Nabbe

**Affiliations:** a Department of General Practice Research Team SPURBO (EA 7479), Faculté de Médecine, Université de Bretagne Occidentale, Brest, France

## Abstract

**Background:** Over the past decade, the amount of digital data created by humans with or without connected tools has grown exponentially. The field of primary care (PC) did not escape this digitization, nor the use of Big Data algorithms. To evaluate the results of Big Data research in PC it seemed useful to identify which algorithms are used.

**Research question:** What are the algorithms used for Big Data research in PC research and how are they described?

**Methods:** Systematic review of the literature according to the recommendations of the PRISMA guide. A search equation using the following MeSH terms ‘big data, data mining, Algorithms, Artificial Intelligence, Machine learning, Deep Learning, Neural Networks Natural Language Processing, general practice, electronic health records, health records’ has been applied to the PUBMED database. After a selection of the titles and article summaries according to the inclusion criteria, the full versions of the eligible articles were read and analysed. Referenced articles of the sources articles were added to the analysis. The algorithms described in the articles were extracted and analysed.

**Results:** In total, 778 articles were identified, 169 were eligible for full reading and 26 articles were finally selected. The algorithms listed in the articles are poorly described. The description is usually limited to a general explanation about how the algorithm works. Seven articles gave a partial description of the algorithm; a logic diagram was given in four articles and the codes in only two. Actually, only one article fully describes the algorithm with its mathematical description, its code and its logic diagram.

**Conclusion:** Big Data algorithms in PC are not satisfactorily described. The lack of reproducibility is not compatible with a consistent scientific approach. Researchers should provide more information about the way they extract and analyse their data to give their readers more confidence in Big Data.
